# Impact of the Q.Clear reconstruction algorithm on the interpretation of PET/CT images in patients with lymphoma

**DOI:** 10.1186/s13550-020-00690-6

**Published:** 2020-08-26

**Authors:** Michał Wyrzykowski, Natalia Siminiak, Maciej Kaźmierczak, Marek Ruchała, Rafał Czepczyński

**Affiliations:** 1 Department of Nuclear Medicine, Affidea Poznań, Poznań, Poland; 2 Department of Endocrinology and Metabolism, Poznan University of Medical Sciences, Poznań, Poland; 3 Department of Hematology and Bone Marrow Transplantation, Poznan University of Medical Sciences, Poznań, Poland

**Keywords:** PET/CT, Lymphoma, Q.Clear, Reconstruction algorithm, Deauville criteria

## Abstract

**Background:**

Q.Clear is a new Bayesian penalized-likelihood PET reconstruction algorithm. It has been documented that Q.Clear increases the SUVmax values of different malignant lesions.

**Purpose:**

SUVmax values are crucial for the interpretation of PET/CT images in patients with lymphoma, particularly when the early and final responses to treatment are evaluated. The aim of the study was to systematically analyse the impact of the use of Q.Clear on the interpretation of PET/CT in patients with lymphoma.

**Methods:**

A total of 280 ^18^F-FDG PET/CT scans in patients with lymphoma were performed for staging (sPET), for early treatment response (iPET), after the end of treatment (ePET) and when a relapse of lymphoma was suspected (rPET). Scans were separately reconstructed with two algorithms, Q.Clear and OSEM, and further compared.

**Results:**

The stage of lymphoma was concordantly diagnosed in 69/70 patients with both algorithms on sPET. Discordant assessment of the Deauville score (*p* < 0.001) was found in 11 cases (15.7%) of 70 iPET scans and in 11 cases of 70 ePET scans. An upgrade from a negative to a positive scan by Q.Clear occurred in 3 cases (4.3%) of iPET scans and 7 cases (10.0%) of ePET scans. The results of all 70 rPET scans were concordant. The SUVmax values of the target lymphoma lesions measured with Q.Clear were higher than those measured with OSEM in 88.8% of scans.

**Conclusion:**

Although the Q.Clear algorithm may alter the interpretations of PET/CT in only a small proportion of patients, we recommend using standard OSEM reconstruction for the assessment of treatment response.

## Background

Q.Clear is a new Bayesian penalized-likelihood iterative positron emission tomography (PET) reconstruction algorithm that can control the background noise textures in images depending on the level of the activity that is installed in some newer PET/CT scanners [1]. The algorithm includes a point spread function based on the relative difference penalty, which is a function of both differences between neighbouring voxels and their sum [2]. Point spread function modelling results in noise suppression, allowing an increase in the number of iterations without background noise, usually noticed in ordered subsets expectation maximization (OSEM) [3], and it is controlled by a penalization factor beta parameter, which is the only input variable under the influence of the user. Compared with the image analysis using OSEM, image analysis using the Q.Clear algorithm resulted in increased maximal standardized uptake values (SUVmax) [2, 4], especially in small lesions such as lung cancer and lymphoma lesions [5]. In the studies reported to date, the increased sensitivity of Q.Clear compared with OSEM has been described both in phantom studies [1] and in certain clinical conditions [6], including malignant lung tumours [7, 8], metastases of non-small cell lung cancer to mediastinal lymph nodes [9] and colon cancer liver metastases [10].

PET/CT with 18F-fluorodeoxyglucose (^18^F-FDG) has been widely used in patients with both Hodgkin lymphoma (HL) and non-Hodgkin lymphoma (NHL) in several stages of management: for precise staging before treatment initiation [11–13] and for early and final assessments of the response to chemotherapy [14]. The treatment response is evaluated by the comparison of the ^18^F-FDG uptake in the lymph nodes or organs affected by lymphoma with the uptake in normal tissues. As it has been recommended by expert panels, the systematic, semi-quantitative assessment of the treatment response involves measurement of ^18^F-FDG uptake expressed by SUVmax in the areas involved and in the reference regions of the liver and mediastinal blood pool (MBPS). The relationship of the measured SUVmax values is scored with the 5-point Deauville scale (DS), named after the place of its approval for clinical practice by the First International Workshop on Interim-PET-Scan in Lymphoma [15]. Scores of the Deauville scale vary between 1 and 5; scores of 1–3 are interpreted as negative, and scores of 4–5 are considered positive.

As the clinical trials that resulted in the introduction of the DS to the international guidelines were based on SUVmax measurements made with the use of the OSEM reconstruction algorithm, we found it necessary to evaluate the possible impact of the novel Q.Clear algorithm on the interpretation of PET/CT images and to determine whether both algorithms might be used alternatively in lymphoma patients. Therefore, the aim of this retrospective study was to analyse the impact of the use of the Q.Clear algorithm for the interpretation of PET/CT images in patients with lymphoma at different stages of the management:

1. During staging before treatment initiation
2. For the early assessment of treatment response
3. For the final assessment of treatment response
4. For the detection of relapse

## Materials

A total of 280 PET/CT scans performed between March 2015 and December 2018 in our institution in 171 consecutive patients with lymphoma were retrospectively analysed. The consecutive scans were assigned to one of the 4 subgroups (each including 70 scans), according to their clinical purpose: PET/CT performed for staging of the disease (sPET), for early treatment response-interim PET/CT (iPET), after the end of treatment (ePET) and when a lymphoma relapse was clinically suspected (rPET). The patients from each subgroup are characterized in Table 1.

**Table 1:** Patient characteristics in the subgroups

PET/CT scan	Number of scans	Female patients	Hodgkin lymphoma patients*	Age range [years]	Median age [years]
sPET	70	33 (47.1%)	34 (48.6%)	6–84	46.5
iPET	70	30 (42.9%)	56 (80.0%)	13–80	43.5
ePET	70	29 (41.4%)	33 (47.1%)	17–83	43.0
rPET	70	44 (62.9%)	33 (47.1%)	9–80	53.5
Total	280	136 (48.6%)	156 (55.7%)	6–84	45.0

*Remaining patients were diagnosed with non-Hodgkin lymphoma

## Methods

Patients referred for PET/CT for staging (sPET subgroup) were scanned 1–21 days before treatment initiation. In the iPET subgroup, PET/CT was performed in accordance with the current guidelines after 2 or 4 courses of chemotherapy according to the diagnosis and treatment regimen and shortly before the next scheduled course of treatment. In the ePET subgroup, PET/CT was scheduled 3–8 weeks after the last course of chemotherapy, which was after 6 weeks in most cases. Patients who had undergone PET/CT scans performed at least 6 months after treatment with the intention of relapse detection or confirmation of remission were included in the rPET subgroup.

All scans were performed with the use of a Discovery IQ scanner (GE Healthcare). Patients were informed about the necessity of fasting and avoiding physical effort for 4–6 h before the examination. The glucose level was evaluated before the injection of the radiopharmaceutical; the upper accepted limit of the glucose level was 10 mmol/l (180 mg/dl). The acquisition was obtained 60 ± 10 min after the injection of 4 MBq/kg ^18^F-FDG. Routine whole-body scans covered the area from the top of the head to the mid-thigh level. The acquisition time was 1.5 min per bed position.

PET images were reconstructed with two algorithms: OSEM and Q.Clear. The OSEM reconstruction was performed with a 70-cm dual field of view (DFOV) into a 256 × 256 matrix with 4 iterations, 12 subsets and 6.4 mm of full width at half maximum (FWHM). Reconstruction with Q.Clear was performed with a *β* parameter of 350, which was selected basing on our own phantom studies [16]. Both PET results after different reconstructions were fused within the same CT image with the following parameters: 1.25-mm layer thickness, 1.375:1 pitch, 50-cm DFOV 50 and 512 × 512 matrix.

Visual interpretation of images and measurement of SUVmax values were performed using the diagnostic workstation AW 4.4 (GE Healthcare), which provides maximum intensity projections (MIPs), multislice PET and CT images, and their fusion—PET/CT.

The MBPS, liver and lymphoma lesions were segmented manually. SUVmax measurements of the liver were obtained using a 3-cm spherical region of interest (ROI), which was inserted in the area with the highest 18F-FDG uptake in the right liver lobe. For MBPS evaluation, a 1-cm ROI was placed over the central area of the aortic arch. In cases of target lesions (lymphoma infiltration), the ROI diameter was adapted to the size of the lesion. If multiple lymph nodes were involved, a focus with the highest 18F-FDG uptake was selected for evaluation (further referred to as the target lesion).

All scans were rated separately by two experienced nuclear medicine physicians. In cases of controversial or equivocal images, the diagnosis was made by a consensus of evaluating physicians. PET/CT scans obtained using both algorithms were compared according to the following clinical criteria:

1. sPET—the clinical stage according to the Lugano classification [17]
2. iPET and ePET—response evaluation expressed as the Deauville score [17]
3. rPET—clinical interpretation of the scan: negative (complete remission) vs. positive (recurrence)

In the study, the SUVmax of the target lesion 2 times higher than that of the liver was defined as DS = 5.

To obtain the DS, the SUVmax quantification method was used.

### Statistical analysis

Statistical analysis was performed using the Statistica software (TIBCO Software Inc.). The Shapiro-Wilk test was performed to verify a normal distribution. For non-normally distributed data, the results are expressed as median values, and the differences were evaluated by the Wilcoxon test. Data with a normal distribution are shown as the mean ± SD, and the analysis was performed using Student’s *t*-paired test; a *p* value less than 0.05 was considered significant.

## Results

### Staging PET/CT

**Fig. 1:**
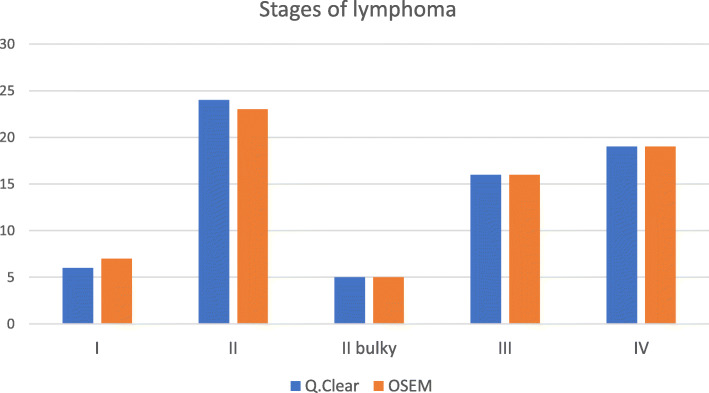
Number of patients with each stage of lymphoma as assessed by two reconstruction algorithms—Q.Clear and OSEM

Among the 70 sPET results, the lymphoma clinical stage was concordantly evaluated in 69 cases (98.6%). In one patient (1.4%) with HL, the Q.Clear algorithm increased the stage from 1 to 2; the upgrade had no significant influence on the management. The precise distribution of stages is presented in Fig. 1.

### Interim PET/CT

A total of 70 PET/CT scans were performed to evaluate the response to chemotherapy. As assessed by the DS score, the results were concordant in 59 cases (84.3%), i.e. the same DS was obtained with both reconstruction methods.

As presented in Table 2, the analysis of PET/CT images with Q.Clear and OSEM showed a discordance of the DS in 11 cases (15.7% cases), and the differences were statistically significant (*p* < 0.001). In 3 patients (4.3%), Q.Clear reconstruction resulted in a change in the DS from 3 to 4, which subsequently led to an upgrade to the positive PET group.

**Table 2:** Deauville scores obtained using Q.Clear and OSEM-interim PET

Deauville score		Q.Clear				
		1	2	3	4	5
OSEM	1	–	–	–	–	–
	2	–	23	3	–	–
	3	–	–	21	3	–
	4	–	–	–	13	5
	5	–	–	–	–	2

Despite conversion to the positive PET group by Q.Clear reconstruction, the treatment strategy in these patients with HL was continued as initially planned.

Each of these three patients underwent another PET/CT examination for the final evaluation of treatment response (ePET). In two of them, a complete metabolic response was confirmed since DS = 2 was scored with the use of both reconstruction methods. In the third patient, ePET showed pathological right external iliac lymph nodes with increased ^18^F-FDG uptake in both reconstruction algorithms. Detection of the new lymph nodes was classified as the progression of the disease, and the patient was qualified for another course of chemotherapy. Therefore, positive iPET with Q.Clear could have correctly converted one patient out of 70 to the worse prognosis group.

### End of treatment PET/CT

Among the 70 ePET scans performed after the completed treatment, concordant results with both algorithms were observed in 59 cases (84.3%). Discrepancies in the DS after using both reconstructive algorithms occurred in 11 cases (15.7%). The detailed DS scores obtained are presented in Table 3.

**Table 3:** Deauville scores obtained using Q.Clear and OSEM after completed treatment

Deauville score		Q.Clear				
		1	2	3	4	5
OSEM	1	–	–	–	–	–
	2	–	27	3	–	–
	3	–	–	25	6	1
	4	–	–	–	1	1
	5	–	–	–	–	6

The observed DS discordances between Q.Clear and OSEM were statistically significant (*p* < 0.001). In 7 patients (10.0%), the use of Q.Clear caused conversion to the positive PET group. Two of these patients, who had been initially diagnosed with stage III lymphoma, were qualified to undergo selective radiation therapy due to positive PET results with remaining high activity in the axillary lymph nodes. In both cases, the follow-up PET/CT examination 3 months after radiotherapy did not show increased ^18^F-FDG uptake in these lymph nodes.

In another patient with elevated ^18^F-FDG uptake in unilateral inguinal lymph nodes (DS = 4 according to Q.Clear and DS = 3 according to OSEM), the decision was made to perform a follow-up PET/CT instead of treatment escalation. The scan obtained 6 months later showed similar uptake in these nodes. The histopathological verification of the nodes confirmed benign inflammatory infiltration with no signs of lymphoma involvement.

**Fig. 2:**
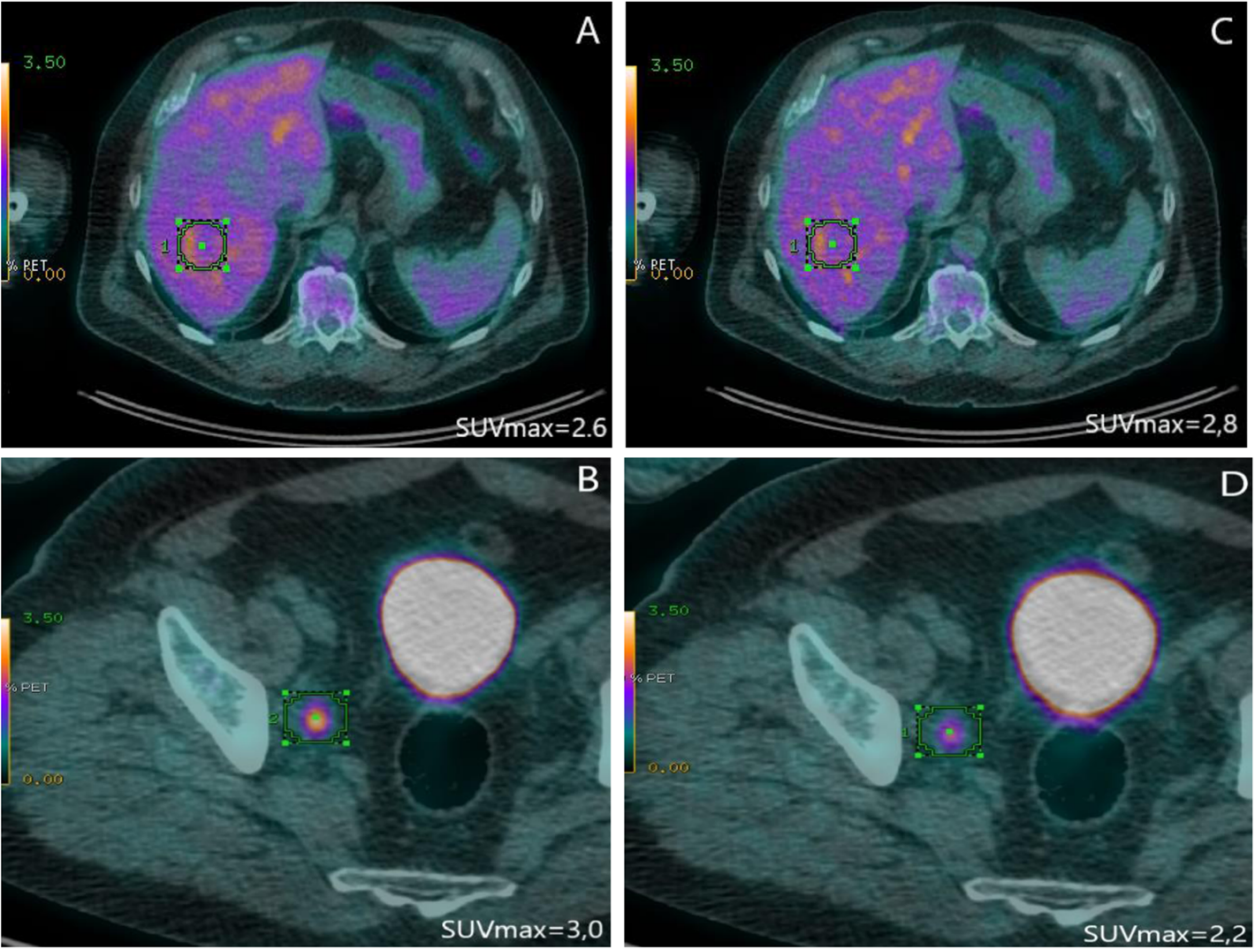
SUVmax values in the liver (**a**) and in the target lesion (**b**) using Q.Clear, and liver (**c**) and the target lesion (**d**) using OSEM

In another 64-year-old patient with NHL, a round iliac lymph node with increased ^18^F-FDG uptake was detected on ePET. Using Q.Clear, the SUVmax was 3.0, which was higher than the liver SUVmax = 2.6. The scan was interpreted as positive (DS = 5 because of a new lesion that was negative on previous scans), and the patient was qualified to undergo the next treatment regimen, which led to the metabolic and morphologic regression of the node. The positive reaction to treatment indirectly confirmed the involvement of the node. However, if OSEM was used, the scan would have been interpreted as negative since the SUVmax value of this node was lower than that of the liver (2.2 vs. 2.8, respectively), which would have led to a conclusion of a negative scan (DS = 3). Adequate images are presented in Fig. 2.

After the analysis of the retrospective results of all DS scores (i.e. both iPET and ePET), it was observed that PET performed with the Q.Clear reconstruction algorithm caused an increase in the DS in 22 cases (15.7%). Concordant results were observed in 118 cases (84.3%). The differences in the DS were statistically significant (*p* < 0.001). In 10 patients (7.1%), the increase in the DS caused conversion to the positive PET group. The difference was also statistically significant (*p* = 0.007), and in 4 patients, it had an effect on treatment strategy: 1 patient was referred for a new chemotherapy course; in the other 2 patients, selective radiotherapy was performed, and 1 patient had a biopsy of lymph nodes.

### Detection of relapse

In the retrospective analysis of 70 rPET scans, all the results were concordant. Scans assessed with the Q.Clear as well as OSEM reconstructive algorithms showed a relapse in 13 cases (18.6%) and complete remission in 57 patients (81.4%).

### Reference regions and target lesion

Additionally, the SUVmax values of reference regions (MBPS and liver) and of target lesions obtained with both reconstruction algorithms were compared at each stage of lymphoma management. In summary, the SUVmax of the MBPS, liver and target lesions of 280 PET/CT examinations were evaluated. Using the Q.Clear algorithm, the SUVmax values of the MBPS were higher in 90 cases (32.1%), equal in 75 (26.8%) and lower in 115 scans (41.1%) than those determined using OSEM. For the liver reference region, the SUVmax values measured with Q.Clear were higher in 75 cases (26.8%), equal in 63 (22.5%) and lower in 142 patients (50.7%). In cases of target lesions evaluated in 223 PET scans, the SUVmax measured with Q.Clear was higher in 198 patients (88.8%) than that determined with OSEM and equal in 25 (11.2%) patients; no cases of a lower SUVmax measured with Q.Clear were recorded.

We evaluated the percentage of small target lesions (defined as smaller than 25 mm) in the series of scans obtained at different stages of lymphoma management in our cohort. The numbers of small target lesions in the subgroups are as follows: sPET 6 out of 70 (8.6%), iPET 48 out of 70 (68.6%), ePET 47 out of 70 (67.1%) and rPET 4 out of 13 (30.1%). The SUVmax values in each group are presented in Table 4.

**Table 4:** SUVmax values measured in smaller (< 25 mm) and larger (≥ 25 mm) target lesions using both reconstruction algorithms

	Target lesion, < 25 mm (Q.Clear)		Target lesion, < 25 mm (OSEM)		Target lesion, ≥ 25 mm (Q.Clear)		Target lesion, ≥ 25 mm (OSEM)		SUVmax ratio*	
	SUVmax, range	Mean, SUVmax	SUVmax, range	Mean, SUVmax	SUVmax, range	Mean, SUVmax	SUVmax, range	Mean, SUVmax	< 25 mm	≥ 25 mm
sPET	3.8–13.0	6.9	2.9–9.6	5.2	3.7–23.6	10.4	3.2–20.7	9.1	**1.33**	**1.14**
iPET	1.2–13.2	3.2	1.1–10.5	2.5	1.4–18.8	2.9	1.1–16.9	2.6	**1.28**	**1.11**
ePET	1.2–14.2	2.8	1.1–11.8	2.4	1.3–10.4	2.7	1.3–7.6	2.4	**1.16**	**1.13**
rPET	4.5–6.6	5.2	3.1–4.8	3.9	4.2–13.8	8.6	3.8–10.9	7.6	**1.33**	**1.13**

*SUVmax ratio—the mean SUVmax measured using the Q.Clear divided by the mean SUVmax of the lesion measured using the OSEM reconstruction algorithm

## Discussion

Several new reconstruction algorithms have been proposed to improve the quality of PET/CT images. One of them, the Q.Clear algorithm, is a valuable diagnostic tool with well-documented utility for the evaluation of lung tumours [7, 9]. Q.Clear increases the detection rate of small PET-positive lesions by providing “truer” SUVmax values compared to other reconstruction algorithms such as OSEM in which the iterative process is stopped before too much noise is introduced. It is of much interest how Q.Clear modifies the interpretation of PET/CT images in other diseases. In the case of lymphoma, it is of particular significance since the measurement of the SUVmax values in the lymphoma foci and in reference regions is a crucial element of interpretation. A modification of the SUVmax measurement methodology can influence final reports and clinical decisions. This retrospective study provides some new insight into the role of Q.Clear in diagnostic PET/CT in patients with lymphoma.

According to Barrington et al., Q.Clear is characterized by higher sensitivity but lower specificity than OSEM [18]. The present study shows that the Q.Clear reconstruction algorithm may influence the SUVmax values of both target lesions and reference regions that may subsequently lead to altered interpretation of the scans in a small proportion of patients. This impact is of particular significance when the Deauville criteria are used since upstaging from a negative (DS = 3 or less) to a positive (DS = 4 or 5) scan may lead to treatment escalation with the administration of highly toxic and costly medication. After the demonstration of the differences in the DS in patients enrolled in our study, the main question is whether images with higher SUVmax values measured with Q.Clear in target lesions truly represent residual lymphoproliferative disease or rather an inflammatory process. A definitive answer to this question would be possible with histopathological verification of the lesions, which was obviously unavailable due to limited anatomic accessibility (mediastinum, abdomen) and suppressed immune competence during or after chemotherapy. We were only able to confirm one case of a false-positive PET result after the use of the Q.Clear algorithm in a patient who presented with suspicious inguinal lymph nodes, while the PET scan after OSEM reconstruction showed a negative result. This may confirm the assumption of the lower specificity of the Q.Clear algorithm. At the same time, we found a case of true-positive ePET result with Q.Clear (false negative using OSEM), which may suggest a slightly higher sensitivity of the Q.Clear algorithm.

Initial clinical studies of the Q.Clear algorithm have demonstrated increased SUVmax values in smaller lesions [5, 6, 19]. Therefore, it was of special interest whether the small size of lymphoma lesions influenced PET/CT interpretation while using Q.Clear. To briefly verify this hypothesis, we divided the target lesions into two subgroups according to their size. As proposed earlier by Kuhnert et al. [20], lesions smaller than and equal to 25 mm were defined as small. We decided to use the threshold of 25 mm as well.

As expected, on iPET and ePET scans, significantly higher rates of small target lesions have been observed than on sPET and rPET. Therefore, this higher representation of small lesions on iPET and ePET scans may be responsible for the upstaging of the Deauville score in a number of patients.

Another important issue related to the use of the novel reconstruction algorithm is its influence on PET/CT image interpretation in patients with lymphoma—a significant increase in SUVmax values was observed in the MBPS, liver and target lesions. Barrington et al. [18] pointed out that the higher selective values of SUVmax in small lesions, e.g. lymph nodes, with none or a minor influence on the uptake of ^18^F-FDG in reference regions, i.e. the MBPS and liver, may lead to false image interpretation. In our study, however, in all four groups of PET scans, the SUVmax values of the MBPS and liver were rather lower when measured with Q.Clear than with OSEM. Therefore, our results are slightly different than previously published observations that showed no difference or even a slight increase in SUVmax in the MBPS and liver regions [18, 21]. Furthermore, Matti et al. did not find any modification in the background signal in their recently published analysis with Q.Clear in different clinical conditions [6]. However, consistent with our data, they reported amplification of the signal of hypermetabolic findings, which led to an increase in the signal-to-noise ratio, improving the overall image quality.

It must be pointed out that despite the slight decrease in the SUVmax in reference regions with the use of Q.Clear, the increase in the DS score was caused in all analysed cases by the increase in the SUVmax values in target lesions, not by the SUVmax alterations in the reference regions.

Some recent studies have compared OSEM with newly implicated reconstruction algorithms. The impact of the point spread function (PSF) (Siemens HD) was analysed in a similar context in a study of Enilorac et al. [22]. The authors reviewed 195 PET/CT scans in patients with diffuse large B cell lymphoma. Despite the difference in the technique, the obtained results were similar to ours. Discordant values of the DS were found in 14% of patients, and a classification change in terms of negativity-positivity was observed in 5% (the respective values in our study were 15.7% and 4.3%). However, it should be highlighted that, as opposed to our study, the authors did not exclude patients with DS = 1. Therefore, these data are not quite directly comparable. Moreover, in contrast to our study, the change in interpretation was not only conversion to positivity. Their algorithm led not only to upstaging to positivity (4 cases) but also to downstaging to negativity in one patient. A study similar to ours was recently performed by Ly et al. comparing the Q.Clear and EARL standards. In their study, 54 PET/CT scans in patients with lymphoma were reviewed [23]. The authors found a discordance between both standards in one third of the patients, and in 5 cases (9.3%), the use of Q.Clear caused conversion to the PET-positive group. Thus, the impact of the Q.Clear algorithm was noted in a larger proportion of patients than in our study. It can be speculated that this difference may be caused by the different beta values: 500 was used in the study by Ly et al. compared to 350 in our paper.

Therapeutic decisions in lymphoma patients are mostly based on clinical guidelines, such as those of the National Comprehensive Cancer Network (NCCN), in which PET/CT examination plays a pivotal role [24, 25]. There is hardly any other disease with such a strong influence of PET/CT on clinical decisions. The interpretation of PET/CT images based on precise criteria such as the DS is of crucial importance. Those criteria and guidelines were developed before the introduction of new reconstruction algorithms such as Q.Clear for PET/CT scanners and were based on previous PET system generations. Commonly used recommendations of scientific societies, including the Lugano classification, are based on numerous large prospective clinical studies [14, 26] in which the routine OSEM reconstruction algorithm was used in all scanners from all manufacturers. Novel reconstruction algorithms, such as Q.Clear, aiming at the improvement of the tumour detection rate or improvement of spatial resolution are very helpful in various clinical conditions. We must be aware, however, of the potential pitfalls caused by this new technology. Nevertheless, the differences between OSEM and Q.Clear, which have been presented in this study, are minor and refer to only some aspects of clinical decision making. They do not allow us to unequivocally acknowledge the new technology as ready for introduction into clinical practice in lymphoma management. Further multicentre studies involving large patient cohorts and long follow-up could be potentially helpful in elucidating the impact of Q.Clear and other innovative reconstruction algorithms on the management in lymphoma.

## Conclusions

According to the presented results and our experience, the routine use of the Q.Clear algorithm alone for therapeutic decisions in patients with lymphoma seems to be uncertain, mainly because of the incompatibility with the current guidelines and recommendations. Therefore, we suggest that the Q.Clear reconstructive algorithm not be used in the evaluation of images for the assessment of treatment response both during and after therapy unless its verification in large-scale clinical trials occurs. Despite no apparent need for the withdrawal of Q.Clear in staging as well as in the detection of relapse, we still suggest the use of the standard OSEM reconstruction algorithm in all stages of management for comparability reasons.

## Data Availability

All data generated and analysed during this study are included in this published article.

## Abbreviations

PET: Positron emission tomography
OSEM: Ordered subsets expectation maximization
^18^F-FDG: 18F-fluorodeoxyglucose
SUV: Standardized uptake value
HL: Hodgkin lymphoma
NHL: Non-Hodgkin lymphoma
MBPS: Mediastinal blood pool
DS: Deauville scale
sPET: PET/CT performed for staging of the disease
iPET: PET/CT performed for early treatment response-interim
ePET: PET/CT performed after the end of treatment
rPET: PET/CT performed when a lymphoma relapse was clinically suspected
NCCN: National Comprehensive Cancer Network
